# Chronic Pain and Substance Use Disorders: A Brief Narrative Review of Genetic, Neurobiological, and Environmental Contributions to Comorbidity

**DOI:** 10.20900/jpbs.20250003

**Published:** 2025-06-10

**Authors:** Pamela N Romero Villela, Emma C Johnson

**Affiliations:** Department of Psychiatry, Washington University School of Medicine, St. Louis MO 63110, United States

**Keywords:** chronic pain, substance use disorders, comorbidity

## Abstract

**Background:**

Chronic pain (CP) and substance use disorders (SUDs) frequently co-occur. This brief review highlights environmental, neurobiological, and genetic sources of comorbidity of CP and SUDs, focused on alcohol, nicotine, cannabis, and opioids.

**Methods:**

A literature search on CP and SUDs was performed using Google Scholar and PubMed. Relevant literature was summarized in a narrative review.

**Results:**

Recent genomic studies reveal that SUDs and CP share a significant portion of genetic variance, and causal inference methods suggest that CP and SUDs have bidirectional effects on one another. CP and SUDs share multiple neurobiological pathways such as the reward and stress systems, with studies implicating important regions such as the insular and anterior cingulate cortex, the ventral tegmental area, and the nucleus accumbens. Environmental risk factors for CP and SUDs include socioeconomic background, education, and broader environmental factors such as neighborhood resources, air quality and greenspace. Social support is also a protective factor against CP and SUD diagnoses and crucial for their successful treatment and remission.

**Conclusions:**

Promising new areas of research underlying CP and SUD comorbidity include female-specific CP conditions and substance use patterns, the role of the immune system in both SUDs and CP, and the rise of large biobanks that will further precision medicine by allowing researchers to jointly model genetic, neurobiological, and socioenvironmental factors underlying their co-occurrence. In summary, CP and SUDs are debilitating conditions with far-ranging consequences for both individuals and communities; investigating their shared etiology will result in better treatments for both.

## INTRODUCTION

Chronic pain (CP) is broadly defined as pain lasting three months or more [1]. CP is common, affecting 33% of the worldwide [2] and 24.3% of the United States (US) population [3]. It is also highly heterogeneous, with a wide range of potential causes, from injury to surgery to neurological disease. Severe CP can impair day to day activities [4], lead to depression [5], and result in long-term physical disabilities [6]. CP also burdens national healthcare and economic systems, costing between $560–$635 billion annually in the US alone [2]. Substance Use Disorders (SUDs) are generally defined as continued, compulsive substance use, even in the face of social and physical consequences, to alleviate uncomfortable feelings when not using the substance [7]. Like CP, SUDs are also relatively common (depending on the substance) and present a major public health burden, costing the US over $13 billion in 2017 alone [8]. According to the Centers for Disease Control, around 14.5% of people in the United States over the age of 12 have a SUD [9]. Individuals suffering from SUDs often use multiple substances (polysubstance use [10]), and SUDs are frequently comorbid with other mental [11] and physical health conditions, including depression [12], cardiovascular disease [13], and chronic pain [14].

CP and SUDs co-occur frequently; studies report that around 40% of individuals suffering CP also met criteria for SUD [15]. Co-occurring pain further complicates SUD recovery; for example, a study [16] found that 20.6% (7.6 million people) of adults in the US with a past year SUD diagnosis reported having pain interfering with their SUD recovery. Therefore, understanding the mechanisms by which CP and SUDs co-occur is important to develop effective treatments for patients suffering from both diagnoses and ensure patients continue being successful in their SUD remission efforts.

**Figure 1.**
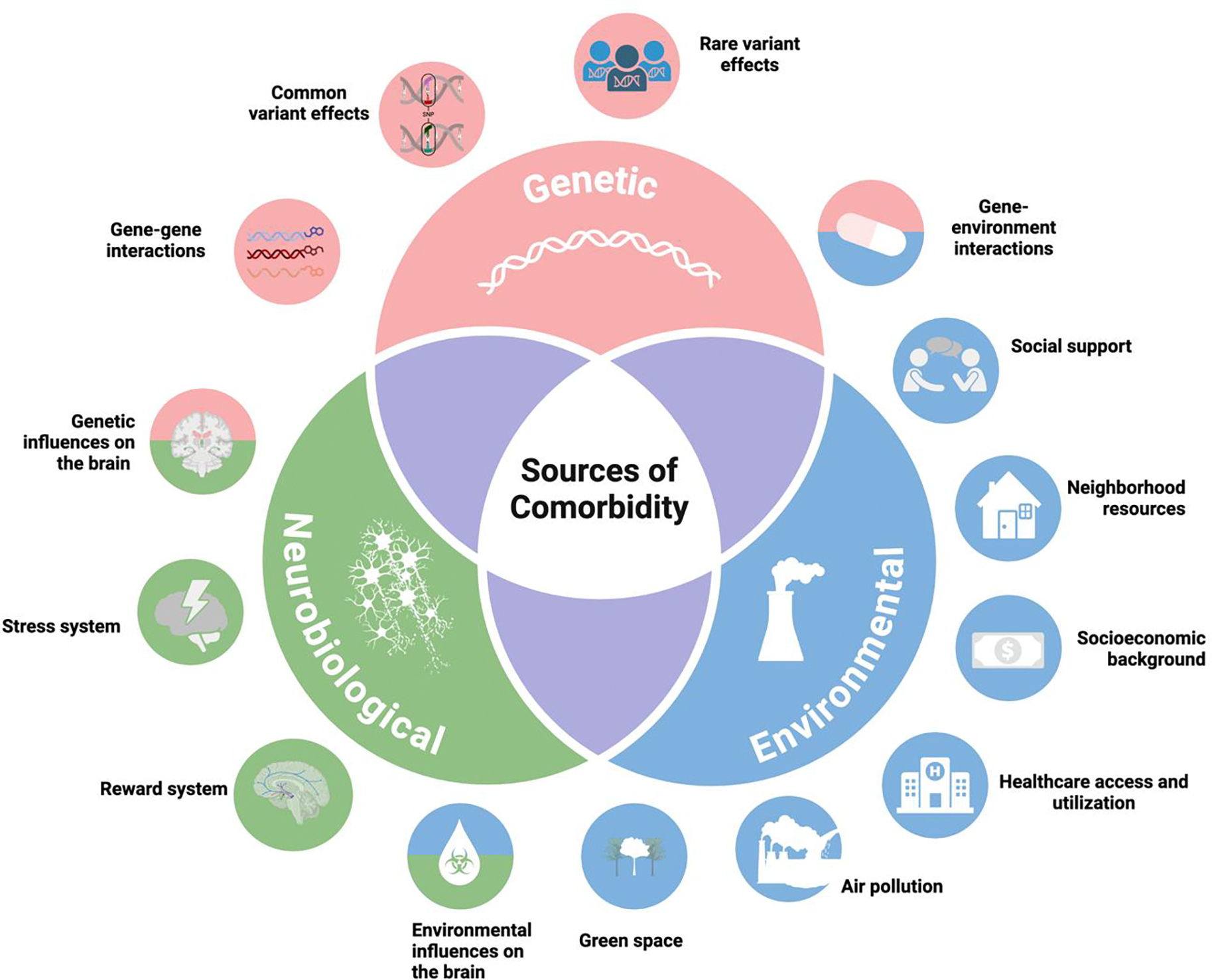
Overview of sources of comorbidity between chronic pain and substance use disorders, and specific shared risk and protective factors.

This narrative review highlights the genetic, neurobiological, and environmental risk and protective factors that are shared between CP and SUDs and may explain their co-occurrence (for overview, see Figure 1). Throughout, the focus is on alcohol, nicotine, cannabis, and opioids, as these are the most used substances in the United States and the most well-studied in terms of their genetic etiology and connections with CP. First, evidence of genetic overlap between CP and SUDs will be reviewed. Next, this review will discuss shared neurobiological mechanisms, followed by environmental sources of CP and SUD comorbidity. Finally, the review will conclude with a summary of key findings.

## COMORBIDITY OF CHRONIC PAIN AND SUBSTANCE USE DISORDERS

Opioid use disorder (OUD) is the most well-known SUD in the context of comorbidity with CP [17]. Opioids are often prescribed to manage pain, and an estimated 3%–4% of adults in the US have had a long-term opioid prescription [17]; approximately 35%–12% of adults using opioids to treat chronic pain will develop opioid use disorder or abuse [14]. Males and younger individuals are at greater risk of developing OUD after being prescribed opioids to treat CP, and those with personal or family history of SUDs are also at increased risk [14]. Other factors that may increase risk include negative affect (i.e., anxiety or depression) and self-reported opioid craving [14]. It is important to note that while opioid prescriptions have decreased in the US in recent years, the rate at which opioid overdose deaths have decreased varies greatly depending on the kind of opioid used. For example, while deaths due to prescription opioids and heroin have generally remained steady or declined since around 2017, overdose deaths due to fentanyl and other synthetic opioids had been increasing sharply until 2023 (the “third wave” of the opioid crisis) [18,19]. For the first time since 2018, total drug overdose deaths decreased from 2022 to 2023, including a decrease in deaths due to synthetic opioids such as fentanyl, which showed a decrease of 2.2% [20]. During that same year, the drop in deaths due to heroin was much sharper, around 33.3% [21]. Further research is needed to understand differential risk for addiction and overdose deaths across opioid subtypes.

While the development of OUD remains a concern in patients prescribed long-term opioid therapy, this concern must be balanced with the need to provide appropriate and effective pain treatment to those experiencing CP. Despite the attention that the opioid crisis has gathered, medical schools continue to lack robust pain management training; what is more, even fewer teach about addiction [22]. Lastly, racial disparities in opioid treatment of CP must also be addressed: despite studies suggesting that Black [23] and Hispanic [24] individuals may experience higher rates of CP and pain intensity, studies suggest that Black individuals are often under prescribed opioids for CP management by over 36% compared to their White counterparts [25]. Gaining a better understanding of risk factors that increase one’s liability to develop OUD after long-term opioid therapy and developing alternative, effective pain management strategies are crucial for improving prevention of OUD and treatment of CP.

Alcohol is one of the most widely used substances across the world [26] and may provide some temporary pain relief thanks to its analgesic effects [27]. However, using alcohol for its analgesic properties may then lead to increased tolerance and a need for greater alcohol consumption to achieve the same effects [28]. While low and moderate alcohol use may provide immediate analgesic effects, prolonged use is associated with worse pain trajectories over time [29]. Individuals with problematic alcohol use are more likely to experience pain than those without problem use [30], and prolonged alcohol use is a leading cause of neuropathy [31]. In addition, depression may serve as a mediator between CP and alcohol use disorder (AUD), as individuals with CP are more at risk for depression [32] and may drink alcohol to alleviate negative feelings (i.e., negative urgency, the tendency to act impulsively or engage in risky behaviors to assuage negative emotional states [33]).

Cannabis is the most widely used illicit substance across the world, with rates of use around 2.5% [34]. In the US in particular, increased legalization of cannabis has led to an uptick in recreational and medicinal use [35]. With increased legalization of both medicinal and recreational cannabis in the US, cannabis has become an alternative pain therapeutic. One study found that 3 in 10 individuals experiencing CP reported using cannabis to treat their pain [36], despite mixed evidence of the true efficacy of cannabis for treating pain. In population-based [37] and veteran samples [38], adults with CP were significantly more likely to have cannabis use disorder (CUD) compared to their non-pain counterparts. However, a cross-sectional study of patients with CP in Germany found that although 29.9% of patients with CP also reached DSM-IV criteria for CUD, the percentage of patients with comorbid CP and CUD dropped to 2.1% after removing positive behavior items (e.g., tolerance, strong desire and withdrawal from cannabis) from the CUD criteria [39], suggesting that CUD diagnoses in patients with CP might be overestimated when using only DSM-IV criteria for CUD but underdiagnosed by physician observations alone.

While tobacco smoking has generally declined in the US, the prevalence of tobacco use in CP patients remains relatively high (e.g., an estimated 35% of individuals with chronic neck or back pain use cigarettes, which is almost 3 times greater than the national average [40]). While tobacco use may provide short-term analgesic properties, nicotine can also increase pain sensitivity; individuals with CP who use tobacco report worse symptoms and greater pain severity than those who do not ingest tobacco [41]. According to a review [42] on tobacco and pain by LaRowe and Ditre, most studies investigating the link between CP and tobacco have been focused on tobacco use rather than tobacco use disorder (TUD). However, Zvolensky et al. [43] found that lifetime CP incidence was higher among individuals with nicotine dependence than non-dependent nicotine users. This study suggests that the comorbidity between CP and SUDs such as TUD merit further investigation, over and above what has been learned by studying co-occurring pain and substance use behaviors.

## GENETIC SOURCES OF COMORBIDITY

Genetic studies of CP and SUDs are useful for identifying biological mechanisms and/or biomarkers underlying both conditions. Additionally, genetic studies can aid in risk stratification and treatment protocols for individuals suffering from CP and at risk for developing an SUD. Genetic variants can also be used to aid in quasi-experimental Mendelian Randomization (MR) studies [44] to estimate causal relationships between CP and SUDs (and vice versa; See Table 1 for definitions of common genetics terms, including MR, used throughout this section).

To our knowledge, there are no traditional genetic twin studies on the comorbidity of CP and SUDs. Modern molecular genetic studies of CP and SUDs have demonstrated a moderate proportion of shared genetic effects. A recent study by Koller et al. [45] investigated the genetic correlation between multi-site chronic pain (MCP, a score summing the number of body sites an individual experiences CP in, ranging from 0–7) and a variety of SUDs. Genetic correlations between MCP and SUDs ranged from r_g_ = 0.20 for opioid use disorder to r_g_ = 0.37 for CUD. These estimates are in line with other studies such as Deak et al. [46], which estimated the genetic correlations between OUD and CP phenotypes to range from r_g_ = 0.22 (last month: headache) to r_g_ = 0.63 (neck/shoulder pain for 3+ months). Similarly, Toikumo et al. [47] found that the genetic correlation between TUD and MCP was r_g_ = 0.36. In short, recent studies suggest moderate genetic correlations between specific SUDs and CP.

One study examined the genetic liability for SUDs broadly by conducting a multivariate genome-wide association study (GWAS) of general addiction liability (addiction-rf) [48]. This study found that genetic liability for the general addiction factor was significantly associated with CP, in line with the shared neurobiological mechanisms between CP and SUDs elucidated by human and animal studies [19,49]. Interestingly, another study [50] found that after controlling for the genetic effects of tobacco use disorder, there remained a significant association between genetic liability for cannabis use disorder (CUD) and musculoskeletal pain. Taken together, these findings suggest that CP may have genetic risk that is shared across SUDs (i.e., general addiction liability) and shared genetic risk that is specific to certain SUDs.

The Koller et al. study mentioned above [45] also performed pleiotropic analyses, which revealed dozens of genetic loci that influence both MCP and SUDs. Specifically, authors identified 25 loci underlying MCP and AUD, 22 for MCP and CUD, and 4 loci for MCP and OUD. However, no pleiotropy was detected at the gene or pathway level, potentially suggesting differential genetic effects within genes and/or small effects spread across multiple genes, thereby limiting the statistical power to detect such effects at the gene level. Future analyses with greater statistical power may identify pleiotropic variants implicating shared neurobiological pathways between CP and SUDs, such as stress regulation. Koller et al. also employed MR, a causal inference method, to investigate the causal relationship between CP and SUDs. Authors found evidence for causal bidirectional relationships between MCP and AUD, CUD, and Problematic Tobacco Use (PTU). However, they noted that the results were stronger for MCP influencing CUD and PTU rather than vice versa. These results are in line with another recent study [51] that found that genetic liability for chronic back pain caused increased alcohol use and smoking quantity and vice versa. Notably, Koller et al. [45] failed to identify causal relationships between MCP and OUD; authors speculated this was due to low power. Therefore, while additional studies are needed, current research suggests significant bidirectional relationships between genetic risk for CP and SUDs.

### Future Directions

Large-scale biobanks such as the All of Us Research Program [52] and the United Kingdom (UK) Biobank [53] have collected data from around 500,000 individuals in the US and UK. These data are rich and include electronic health records, health and behavior surveys, accelerometers, and, notably, whole genome data. Until recently, datasets with rare genetic variant information available had been scarce due to limitations in sequencing technologies and high costs; accordingly, genetic studies of rare variants underlying the co-occurrence of CP and SUDs are limited [54]. Now, these large-scale biobanks have rare variant information for hundreds of thousands of individuals, enabling rare variant studies of both diseases. Studies of rare variants might implicate novel biological pathways or elucidate the core genes involved in CP-SUD comorbidity, given that rare variants are more likely to reside within essential genes and gene pathways [55]. Genetic interaction (gene-gene and gene-environment) studies investigate how a genetic factor’s effect depends on another genetic or environmental factor. To our knowledge, no studies investigating gene-gene or gene-environment interactions underlying CP-SUD co-occurrence have been reported. (See Table 1 for definitions of common genetics terms.) Both rare variant and genetic interaction studies can improve treatment of CP-SUD comorbidity by contributing to personalized treatment strategies depending on an individual’s genetic makeup (rare variants being particularly important for individual risk prediction) and their environment. Moreover, given the diversity and varied presentation of chronic pain conditions and substance use disorders, future studies using deeply phenotyped samples might be better equipped to pinpoint the genes and biological pathways underlying the co-occurrence of certain types of CP and SUDs (e.g., musculoskeletal pain and cannabis use disorder). Lastly, studying genetic variability in individual responses to drugs or treatments, or pharmacogenetics, may be key to understanding why some people who are prescribed opioids to treat CP develop OUD, while others who are prescribed opioids never develop an addiction. For example, one GWAS found a variant near the *OPRM1* gene that moderated therapeutic methadone dose in individuals of African ancestry [56]. Future studies of cases with CP who develop SUDs compared to controls with CP who do not develop SUDs may shed more light on genetic variation underlying sensitivity to substances following chronic pain. In short, these rich datasets with genotypic, phenotypic, pharmacological, and medical information will allow for modeling both biological (rare and common genetic factors, biomarkers) and environmental (social determinants of health, trauma and stress) factors (and their interactions) in predictions of risk, consistent with recent work that showed that including social determinants of health into machine learning models improved the prediction of high-impact chronic pain [57]. Given the large amount of heterogeneity in the risk factors, clinical manifestation, and symptom trajectory of both CP and SUDs, precision medicine approaches are needed to provide the most effective treatments.

## SHARED NEUROBIOLOGICAL MECHANISMS

### Brain Systems

**Figure 2.**
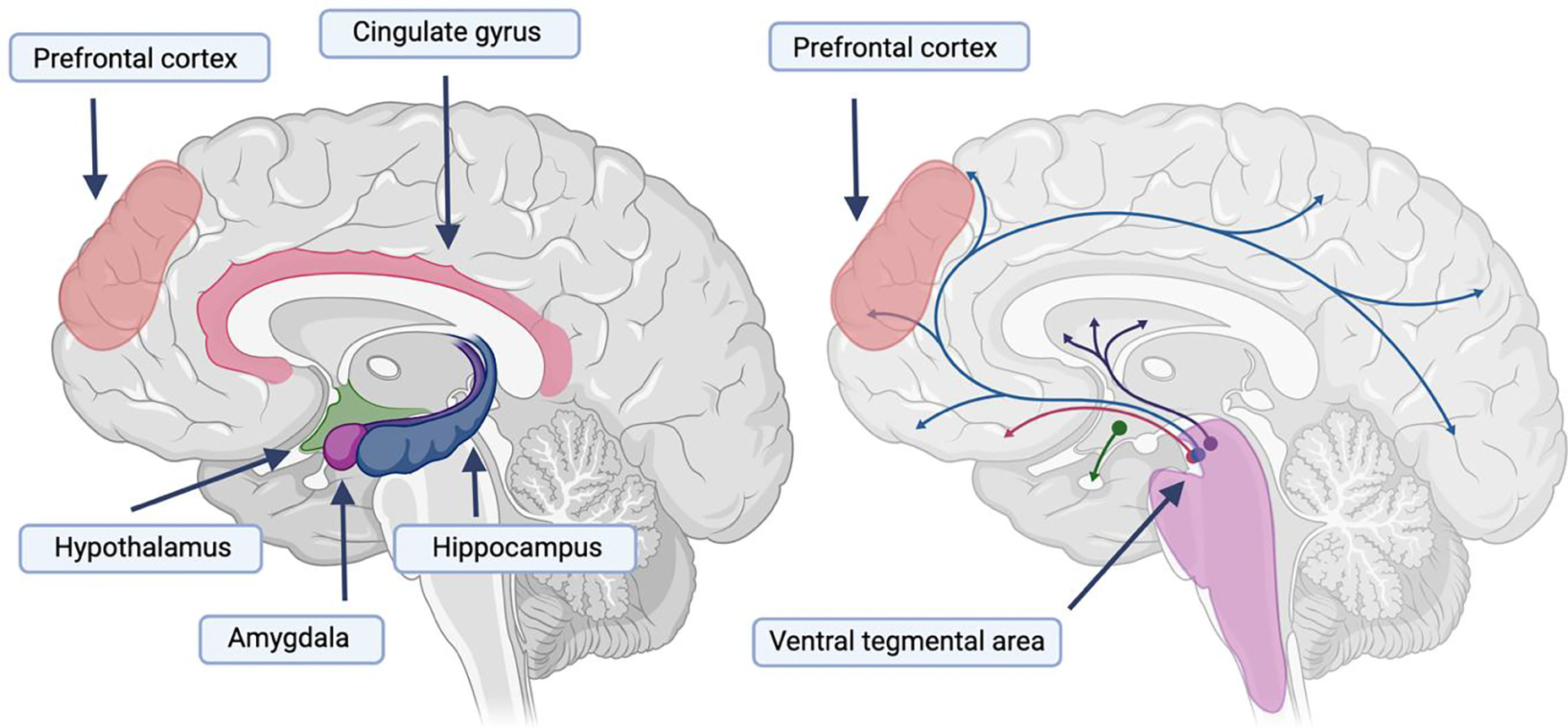
The stress and reward systems associated with both chronic pain and substance use disorders. On the left panel, we show some of the major brain regions associated with responding to stress, including the hypothalamus, amygdala, hippocampus, and prefrontal cortex. On the right, we show dopaminergic projections in the brain associated with the brain’s reward system and highlight the projections starting in the ventral tegmental area and going to the prefrontal cortex. Both systems have been implicated in SUDs and CP.

The stress and reward systems are complex, interconnected brain systems responsible for regulating emotion [58,59], motivation [60,61], and behavior [62,63]. The stress system is located both within the central nervous system (i.e., the brain and spinal cord) and peripheral organs [64]. Within the brain, the structures involved in the stress system include the hypothalamus, pituitary, and adrenal glands (collectively known as the HPA axis); the stress system is additionally composed of the hippocampus, amygdala, and prefrontal cortex (see Figure 2). When individuals experience CP, their stress system is oftentimes chronically activated [65]. In addition, Vachon-Presseau et al. [65] found that pain intensity in individuals with chronic back pain was associated with reduced hippocampal volume [NO_PRINTED_FORM]. Individuals suffering from a SUD also suffer from heightened, prolonged stress [66]. Therefore, given the importance of the stress system for both CP and SUDs, it is likely also a crucial neurobiological factor underlying the CP-SUD comorbidity.

The reward system responds and processes rewarding stimuli, leading to positive reinforcement and pleasurable feelings [59,63]. The reward system is key to learning and motivation. Some of the main brain areas involved in the reward system that will be highlighted here include the ventral tegmental area (VTA), nucleus accumbens, prefrontal cortex, amygdala, and hippocampus. Extensive research has implicated the VTA and nucleus accumbens in the reward and learning processes underlying SUDs [67]. The VTA resides in the midbrain and is involved in learning, memory, stress regulation, emotional processing, alongside sleep and alertness [68]. Dopaminergic activation of the VTA has been found to provide pain relief to mice experiencing models of neuropathic chronic pain [19]. Similarly, in a rat model for chronic inflammatory pain, Ezzatpanah et al. [69] found that blocking the orexin receptors in the VTA led to a dose-dependent reduction in formalin-induced biphasic pain. Interestingly, CP may increase inhibitory neurons that project unto the VTA dopaminergic neurons, thereby making these neurons less excitable and leading to other conditions such as anhedonia [70], which is a psychological condition characterized by a significant decrease in interest in things or activities that were once rewarding [71]. Anhedonia is typical among individuals suffering from substance abuse and significantly impacts the chances of relapse among individuals in remission [72].

Finally, emotion is a key feature of an individual’s personal experience of CP and/or substance use. Key brain areas involved in emotional processing of stimuli include the insular and cingulate cortex. The insular and cingulate cortex are located within the lateral sulcus (i.e., Sylvian fissure) [73] and are key areas of the brain for the process of stimuli, including external stimuli and internal body sensations that might be perceived as painful [74]. These areas are also critical in forming an individual’s emotional and subjective perception of such stimuli [73]. The insular cortex has also been implicated in SUDs; specifically, during abstinence in AUD, and with an individual’s cues and responses to alcohol during abstinence [75]. Animal models further suggest shared neurobiological mechanisms underlying CP and SUD comorbidities. For example, the anterior cingulate cortex has been implicated in both socially and alcohol-mediated pain sensitivity (i.e., hyperalgesia). Lastly, tobacco use has been associated with reduced anterior cingulate and insular cortex in patients with comorbid psychiatric disorders [76], further highlighting how comorbid SUD and CP conditions might interact to challenge treatment.

### Neurotransmitters and Receptors

Differential dopamine signaling and overall dysregulation of the reward and stress systems has been implicated for both CP and AUD (see Figure 2). There are two types of dopamine receptor release patterns, phasic and tonic. Phasic dopamine release is characterized by quick bursts of dopamine release and primarily activates D1 dopamine receptors, while tonic dopamine release is characterized by a continuous dopamine release and mostly involves D2 dopamine receptors. Chronic alcohol exposure leads to decreased phasic dopamine release and increased tonic levels of dopamine, a process hypothesized to underlie tolerance observed among individuals with AUD, such that one needs to consume more alcohol to experience the same effects [77]. The phasic-tonic dopaminergic system is similarly altered in the chronification of pain via a reward deficit state [77]. There are also several overlapping mechanisms in the stress system, including corticotropin-releasing factor receptor 1, whose activation in the hypothalamus and amygdala is associated with stress and anxiety during alcohol withdrawal (leading to increased alcohol consumption to alleviate these symptoms) [78]. Corticotropin-releasing factor receptor 1 is also associated with increased nociception and the chronification of pain [77].

### Neuropeptides

Neuropeptides are chemical messengers released by neurons [79]. Within the brain, neuropeptides can modulate the activity of neurotransmitters; peripherally, neuropeptides can act as hormones and regulate various bodily functions, thereby acting over larger distances than neurotransmitters [80]. A promising neuropeptide to target for CP and SUD comorbidity is orexin. Orexin is a neuropeptide exclusively produced in the hypothalamus [81]. Studies have found that orexin may be a useful tool to reduce inflammation in CP [82], modulate pain transmission, and treat diabetes-mediated pain sensitivity (i.e., hyperalgesia) [83]. For SUDs, orexin has been associated with reward-seeking behavior in animal studies of food, morphine, and cocaine [84]. For example, one study found that orexin gene expression was downregulated in rats who were exhibiting behaviors consistent with acute alcohol withdrawal [85]. Interestingly, in models of OUD, blocking orexin-1 receptors decreased oxycodone self-administration in rats [86]. Moreover, in 2018, the National Institute on Drug Abuse (NIDA) named orexin antagonists as one of the top ten mechanisms with “highest probability of a path to FDA approval for the treatment of some aspect of OUD in the near term” [87]. In short, recent studies have highlighted orexin as a promising target for CP-SUD comorbidity.

### Future Directions

Collectively, studies demonstrate that CP and SUDs share significant neurobiological mechanisms. Together, these shared genetic and neurobiological vulnerabilities lead to increased risk for both conditions. Therefore, understanding how shared neurobiological pathways underlying CP and SUD is important to understand the etiology of their co-occurrence and to develop better treatments for their comorbidity.

Furthermore, multiple studies have linked the immune system to substance use behaviors [88,89] and CP [90]. CP lacks major biomarkers, making diagnosis and treatment development difficult. Raffaeli et al. [91] recently showcased promising results that suggest the Mu opioid receptor on B lymphocytes can be used as a biomarker for CP. Similarly, ongoing phase III clinical trials [92] show that vaccine treatments for SUDs such as cocaine use disorder and TUD might be on the horizon. Immunotherapies for SUDs would be beneficial given their specificity and prolonged effects, providing potentially lifesaving treatment for conditions (e.g., cocaine use disorder) that have no currently approved treatment. Therefore, understanding how the immune system is related to CP and SUDs can further characterization of both CP and SUDs and provide rich avenues of research to develop novel treatments for these challenging conditions.

## ENVIRONMENTAL SOURCES OF COMORBIDITY

### Socioeconomic Background and Access to Healthcare and Treatment

Individuals with lower socioeconomic status (SES) are 1.32 times more likely to experience CP [93]; moreover, their pain is more severe on average than individuals with higher SES [94]. These individuals are also more likely to suffer from a SUD [95]. These associations are likely due to a combination of factors, including occupational hazards and working conditions, limited access to high quality healthcare, comorbid mental health conditions, and increased financial stress [94,96,97]. Lower SES is often accompanied by limited access to healthcare or poor-quality care, which is in turn associated with more severe pain and longer pain duration [3,94]. CP is already challenging to treat, with one study estimating the recovery rate at only 5.4% [98], and limited access to quality healthcare makes treatment even more difficult. Lower SES is also a risk factor for homelessness, which in turn increases the risk of CP and SUDs. One study in the UK found that almost two thirds of individuals experiencing homelessness suffered from CP [99]; similarly, another study estimated two out of every three people experiencing homelessness has a lifetime history of an SUD [100]. A study from Vogel and colleagues suggests that this comorbidity leads to worse treatment outcomes, finding that homeless individuals who reported daily substance use were less likely to receive professional treatment and prescribed medication for their chronic pain [99].

### Neighborhood Risk Factors

Neighborhood-level factors also contribute to the risk of developing CP and SUDs [101], however, to our knowledge, no study has examined neighborhood-level risk factors leading to *co-occurring* CP and SUDs conditions. For example, community violence exposure has been associated with the development of problematic cannabis use in adolescents [102]; similarly, neighborhood instability (a score that aggregates multiple factors such as the percentage of vacant/rental households) increases risk for cannabis use disorder [103]. Violence in a community increases stress, leads to reduced use of urban spaces and can increase feelings of social isolation, and may worsen experiences of chronic pain [104]. Studies have found that people living in areas of greater material deprivation, air pollution, and reduced greenspace are at greater risk for CP [96,105,106] and reduced success in SUD prevention and remission efforts [107–109].

### Social Support and Its Protective Effects

Social support can be conceptualized as the emotional social assistance and comfort an individual receives from others [110]. Social support has been found to be an important protective factor against the stress response to pain [111,112]. Similarly, increased social support increases the life satisfaction [113] and probability of recovery [114,115] for individuals suffering from a SUD. While research investigating the effect of social support on individuals with co-occurring CP and SUD is more limited, a recent study by Benville et al. [116] showed that social support improves both non-cancer CP and opioid use disorder outcomes. However, authors note that individuals suffering from both CP and opioid use disorder on buprenorphine reported significantly less social support, highlighting important intricacies in the comorbidity of CP and SUDs and patients that might be at higher risk for medical complications if current support systems are insufficient. Further underscoring the importance of social support in the comorbidity of CP and SUD, it has been previously reported that individuals suffering from CP are at higher risk of experiencing loneliness, which is linked to increased risk of substance abuse. Therefore, social support is an important factor mediating the relationship between CP and SUDs.

### Other Individual Risk Factors

Demographic characteristics associated with variation in incidence of CP or SUD include sex, race, and gender. For example, females are more likely to report CP than males (25.4% of females compared to 23.2% of males) [3]. Conversely, males have higher diagnosis rates of a SUD [117], while females are typically underdiagnosed with a SUD [118]. In addition, members of underrepresented racial groups, including American Indian/Alaska Native and multiracial individuals, have the highest rates of past year SUDs compared to other racial groups [119]. Like SUDs, CP is more prevalent among American Indian/Alaska Native individuals, followed by White non-Hispanic and then multi-racial non-Hispanic individuals [3]. While most opioid overdose deaths are among White individuals, the rate of drug overdoses is concerningly increasing among Hispanic and Asian groups [120]. While data is limited, research suggests that gender and sexual minority individuals have higher SUD and CP rates than heterosexual or cisgender individuals [121].

### Future Directions

Given the greater prevalence of CP [122–124] and recent changes in substance use patterns in women, including significant increases in alcohol drinking since the COVID-19 pandemic [125,126], research is needed into the mechanisms of comorbidity and consequences for treatment and recovery specifically for women. For example, a recent study [127] revealed phenotypic and genetic associations between a female-specific pain condition, endometriosis, and depression, anxiety, and eating disorders, but no such studies have focused on female-specific pain and SUDs, to our knowledge.

## DISCUSSION

Chronic pain (CP) and substance use disorders (SUDs) are serious health conditions that frequently co-occur. In this review, we have synthetized three major sources of comorbidity, namely, genetic, neurobiological, and environmental. Recent studies report evidence for a shared genetic etiology between these conditions, with moderate genetic correlations between CP and SUDs (ranging from r_g_ = 0.2 (between multi-site chronic pain and OUD [45]) to r_g_ = 0.63 (chronic neck/shoulder pain and OUD [46])). Other studies have shown that CP shares genetic risk with generalized addiction liability [48], as well as sharing genetic risk with specific substances such as cannabis [50]; future studies should leverage methods such as genomic structural equation modeling [128] to further parse genetic risk for CP that is shared across SUDs from genetic risk for CP that is substance-specific. Lastly, MR analyses reveal that CP and SUDs have bidirectional, causal relationships (although there is stronger evidence for CP causing SUDs) [45], further highlighting the complex interplay between these conditions. Therefore, while most studies have focused on how CP might lead to SUDs, the alternative also occurs and needs to be further investigated. Exciting new avenues of research for the genetic underpinnings of CP and SUD are on the horizon thanks to large-scale biobanks with whole genome sequencing data [53,129]. First, it is now possible to conduct well-powered rare variant studies of disease. Rare variant studies will be critical to identify large effect loci important to individual risk prediction and treatment of CP and SUDs. Finally, gene-gene and gene-environment interaction studies will further elucidate how individual genetic factors work together across the whole genomic and environmental landscape to influence individual risk, trajectory, and treatment of CP and SUDs.

CP and SUDs also have multiple overlapping neurobiological mechanisms. First, CP and SUDs are both associated with the stress [49,65] and reward brain systems [63], including the insular and cingulate cortex, prefrontal cortex, and ventral tegmental area, all areas involved in reward and stimuli processing systems. Dopamine and corticotropin-releasing factor receptor 1 are key molecules implicated in both CP [19,130] and SUDs [131,132] through their roles in the reward and stress systems. While genome-wide studies have not yet implicated specific biological pathways shared by CP and SUD, future studies with larger samples may elucidate the extent to which shared genetic liability is enriched in specific cell types, tissues, and pathways, bridging genetic evidence with neurobiological hypotheses. Two additional key areas of further research involve orexin, a neurotransmitter produced in the hypothalamus but with far-ranging modes of action across peripheral body systems. Animal and human studies of orexin have yielded promising results for its ability to manage singular CP [82,83,133] or SUD [85,134,135] diagnoses. Additional research is needed to determine how orexin can be targeted to treat concurrent CP and SUD diagnoses. Like orexin, therapies that have broad targets might be beneficial for both CP and SUD. In fact, immune therapies for individual CP and SUD diagnoses are emerging. Future studies should investigate immune-mediated treatments for individuals suffering from both CP and SUDs.

Lastly, environmental factors underlying CP-SUD comorbidity were reviewed. Socioenvironmental factors that contribute to their co-occurrence include socioeconomic background [93,95] and healthcare access [136], as well as neighborhood-level factors such as neighborhood vacancy rates [101] (although further research is needed to fully understand the influence of neighborhood-level factors on the comorbidity of CP and SUDs, rather than considering risk for each condition separately). Risk of CP and SUDs also vary by individual demographic characteristics, including race, gender and sex [137,138]. Social support is a robust protective factor for both CP and SUDs; moreover, social support is essential for successful treatment of both CP and SUDs. Given the importance of sociobiological factors such as sex in CP and SUD incidence, treatment, and recovery, future research should also focus on understudied CP conditions such as female-specific CP (e.g., endometriosis) and its relationship with SUDs. In short, there are multiple sources of genetic, neurological, and psychosocial comorbidity for CP and SUDs.

## CONCLUSIONS

In summary, chronic pain (CP) and substance use disorders (SUDs) are debilitating and heterogeneous conditions that are difficult to treat and often co-occur. CP and SUDs share multiple sources of biological and environmental risk factors. Genetic studies point to significant shared genetic influences on CP and SUDs, highlighting the role of genetics in their co-occurrence. Genetically informed causal inference studies also highlight the fact that CP and SUDs can have bidirectional relationships. CP and SUDs share multiple neurobiological pathways, including the insular and cingulate cortex, prefrontal cortex, and ventral tegmental area, all areas involved in reward and stimuli processing systems. Socioenvironmental factors that contribute to the co-occurrence of CP and SUDs include socioeconomic background, race, gender, and neighborhood composition such as neighborhood vacancy rates. Social support is a robust protective factor against both CP and SUDs; moreover, social support is essential for successful treatment of both CP and SUDs. In short, CP-SUD comorbidity is complex; novel precision medicine models that account for the wide variety of socioenvironmental and biological factors underlying their co-occurrence will improve their prevention, diagnosis, and treatment.

## Figures and Tables

**Table 1.** Common genetics terms and their definitions.

Genetics Term	Definition

Genetic liability	An individual’s genetic susceptibility for a trait.
Genetic correlation	A measure of the degree to which the effects of genetic factors are shared between two traits.
Pleiotropy	When a variant or gene influences multiple traits.
Genetic locus	genetic marker or gene’s specific, fixed position on a chromosome.
Genetic variant	A change in DNA sequence.
Deep phenotyping	Comprehensive and precise measurement of a trait to capture the full variation and granularity of a trait.
Common variant	A genetic variant that is relatively frequent in a population, typically with a prevalence of 1% or more.
Rare variant	A genetic variant that is relatively rare in a population; a variant with occurrence under 1% is typically considered rare.
Gene-gene interaction	When the effect of one genetic variant or gene depends on another variant or gene.
Gene-environment interaction	When the effect of one genetic variant or gene depends on an environmental factor or exposure.

## Data Availability

No data were generated from the study.

## ABBREVIATIONS

CP: chronic pain
US: United States
SUDs: Substance use disorders
OUD: opioid use disorder
AUD: alcohol use disorder
CUD: cannabis use disorder
TUD: tobacco use disorder
MCP: multisite chronic pain
GWAS: genome-wide association study
addiction-rf: addiction risk factor
MR: mendelian randomization
PTU: problematic tobacco use
HPA axis: hypothalamus, pituitary, and adrenal glands as part of the brain’s stress system
UK: United Kingdom

## References

[1] Anwar K Pathophysiology of pain. Disease-a-Month. 2016;62(9):324–9. 27329514 10.1016/j.disamonth.2016.05.015

[2] Cohen SP, Vase L, Hooten WM. Chronic pain: An update on burden, best practices, and new advances. Lancet. 2021;397(10289):2082–97. 34062143 10.1016/S0140-6736(21)00393-7

[3] Lucas JW, Sohi I. Chronic Pain and High-Impact Chronic Pain Among U.S. Adults, 2023. 2024; Available from: https://stacks.cdc.gov/view/cdc/169630. Accessed on 30 Mar 2025. 10.15620/cdc/169630 PMC11726267 39751180

[4] van den Berg-Emons RJ, Schasfoort FC, de Vos LA, Bussmann JB, Stam HJ. Impact of chronic pain on everyday physical activity. Europ J Pain. 2007;11(5):587–93. 10.1016/j.ejpain.2006.09.003 17107818

[5] Meda RT, Nuguru SP, Rachakonda S, Sripathi S, Khan MI, Patel N. Chronic Pain-Induced Depression: A Review of Prevalence and Management. Cureus. 2022;14(8):e28416. 36171845 10.7759/cureus.28416 PMC9509520

[6] Blyth FM, Van Der Windt DA, Croft PR. Chronic Disabling Pain: A Significant Public Health Problem. Am J Prev Med. 2015;49(1):98–101. 26094230 10.1016/j.amepre.2015.01.008

[7] Volkow ND, Blanco C. Substance use disorders: a comprehensive update of classification, epidemiology, neurobiology, clinical aspects, treatment and prevention. World Psychiatry. 2023;22(2):203. 37159360 10.1002/wps.21073 PMC10168177

[8] Peterson C, Li M, Xu L, Mikosz CA, Luo F. Assessment of Annual Cost of Substance Use Disorder in US Hospitals. JAMA Netw Open. 2021;4(3):e210242. 33666661 10.1001/jamanetworkopen.2021.0242 PMC7936257

[9] Substance Use & Substance Use Disorders | CDC Yellow Book 2024. Available from: https://wwwnc.cdc.gov/travel/yellowbook/2024/additional-considerations/substance-use. Accessed on 30 Mar 2025.

[10] Crummy EA, O’Neal TJ, Baskin BM, Ferguson SM. One is not enough: Understanding and modeling polysubstance use. Front Neurosci. 2020;14:541662. 10.3389/fnins.2020.00569 PMC7309369 32612502

[11] Schuckit MA. Comorbidity between substance use disorders and psychiatric conditions. Addiction. 2006;101(Suppl 1):76–88. 16930163 10.1111/j.1360-0443.2006.01592.x

[12] Hasin D, Kilcoyne B. Comorbidity of psychiatric and substance use disorders in the United States: Current issues and findings from the NESARC. Curr Opin Psychiatry. 2012;25(3):165–71. 22449770 10.1097/YCO.0b013e3283523dcc PMC3767413

[13] Gan WQ, Buxton JA, Scheuermeyer FX, Palis H, Zhao B, Desai R, Risk of cardiovascular diseases in relation to substance use disorders. Drug Alcohol Depend. 2021;229:109132. 34768052 10.1016/j.drugalcdep.2021.109132

[14] Martel MO, Shir Y, Ware MA. Substance-related disorders: A review of prevalence and correlates among patients with chronic pain. Prog Neuropsychopharmacol Biol Psychiatry. 2018;87:245–54. 28669582 10.1016/j.pnpbp.2017.06.032

[15] Tetsunaga T, Tetsunaga T, Nishida K, Kanzaki H, Misawa H, Takigawa T, Drug dependence in patients with chronic pain: A retrospective study. Medicine. 2018;97(40):e12745. 30290690 10.1097/MD.0000000000012748 PMC6200516

[16] Manhapra A, Stefanovics EA, Rhee TG, Rosenheck RA. Persistence of significant pain interference following substance use disorder remission: Negative association with psychosocial and physical recovery. Drug Alcohol Depend. 2022;232:109339. 35121202 10.1016/j.drugalcdep.2022.109339

[17] Dowell D, Haegerich TM, Chou R. CDC guideline for prescribing opioids for chronic pain—United States, 2016. JAMA. 2016;315(15):1624–45. 26977696 10.1001/jama.2016.1464 PMC6390846

[18] Understanding the Opioid Overdose Epidemic | Overdose Prevention | CDC. 2025. Available from: https://www.cdc.gov/overdose-prevention/about/understanding-the-opioid-overdose-epidemic.html. Accessed on 19 May 2025.

[19] Huang S, Zhang Z, Gambeta E, Xu SC, Thomas C, Godfrey N, Dopamine inputs from the ventral tegmental area into the medial prefrontal cortex modulate neuropathic pain-associated behaviors in mice. Cell Rep. 2020;31(12):107812. 32579938 10.1016/j.celrep.2020.107812

[20] U.S. Overdose Deaths Decrease in 2023, First Time Since 2018. Hyattsville (US): National Center for Health Statistics; 2024.

[21] Garnett M, Miniño A, Joyce M, Driscoll A, Valenzuela C. Drug overdose deaths in the United States, 2003–2023. National Vital Statistics Reports. 2024;73(4).

[22] Volkow ND, Blanco C. The changing opioid crisis: development, challenges and opportunities. Mol Psychiatry. 2021;26(1):218–33. 32020048 10.1038/s41380-020-0661-4 PMC7398847

[23] Strath LJ, Sorge RE. Racial differences in pain, nutrition, and oxidative stress. Pain Ther. 2022;11(1):37–56. 35106711 10.1007/s40122-022-00359-z PMC8861224

[24] Yang Y, Reid MC, Grol-Prokopczyk H, Pillemer K. Racial-ethnic disparities in Pain intensity and interference among middle-aged and older U.S. Adults. J Gerontol A Biol Sci Med Sci. 2022;77(2):e74–81. 34265049 10.1093/gerona/glab207 PMC8824568

[25] Morden NE, Chyn D, Wood A, Meara E. Racial inequality in prescription opioid receipt—role of individual health systems. N Engl J Med. 2021;385(4):342–51. 34289277 10.1056/NEJMsa2034159 PMC8402927

[26] Alcohol Research Report. Rockville: National Institute on Drug Abuse; 2023.

[27] Thompson T, Oram C, Correll CU, Tsermentseli S, Stubbs B. Analgesic effects of alcohol: A systematic review and meta-analysis of controlled experimental studies in healthy participants. J Pain. 2017;18(5):499–510. 27919773 10.1016/j.jpain.2016.11.009

[28] De Aquino JP, Sloan ME, Nunes JC, Costa GPA, Katz JL, de Oliveira D, Alcohol use disorder and chronic pain: An overlooked epidemic. Am J Psychiatry. 2024;181(5):391–402. 38706339 10.1176/appi.ajp.20230886 PMC11521207

[29] Zale EL, Maisto SA, Ditre JW. Interrelations between pain and alcohol: An integrative review. Clin Psychol Rev. 2015;37:57–71. 25766100 10.1016/j.cpr.2015.02.005 PMC4385458

[30] Brennan PL, Schutte KK, Moos RH. Pain and use of alcohol to manage pain: prevalence and 3-year outcomes among older problem and non-problem drinkers. Addiction. 2005;100(6):777–86. 15918808 10.1111/j.1360-0443.2005.01074.x

[31] Chopra K, Tiwari V. Alcoholic neuropathy: Possible mechanisms and future treatment possibilities. Br J Clin Pharmacol. 2012;73(3):348–62. 21988193 10.1111/j.1365-2125.2011.04111.x PMC3370340

[32] Bair MJ, Robinson RL, Katon W, Kroenke K. Depression and pain comorbidity: a literature review. Arch Intern Med. 2003;163(20):2433–45. 14609780 10.1001/archinte.163.20.2433

[33] Cyders MA, Smith GT. Emotion-based dispositions to rash action: Positive and negative urgency. Psychol Bull. 2008;134(6):807–28. 18954158 10.1037/a0013341 PMC2705930

[34] World Health Organization. Alcohol, Drugs and Addictive Behaviours. Available from: https://www.who.int/teams/mental-health-and-substance-use/alcohol-drugs-and-addictive-behaviours/drugs-psychoactive/cannabis. Accessed on 31 Mar 2025.

[35] Zellers SM, Ross JM, Saunders GRB, Ellingson JM, Anderson JE, Corley RP, Impacts of recreational cannabis legalization on cannabis use: A longitudinal discordant twin study. Addiction. 2023;118(1):110–8. 36002928 10.1111/add.16016 PMC10086942

[36] Bicket MC, Stone EM, Mcginty EE. Use of cannabis and other pain treatments among adults with chronic pain in US States with medical cannabis programs. JAMA Netw Open. 2023;6(1):e2249797. 36607641 10.1001/jamanetworkopen.2022.49797 PMC9857553

[37] Hasin DS, Shmulewitz D, Cerdá M, Keyes KM, Olfson M, Sarvet AL, U.S. adults with pain, a group increasingly vulnerable to nonmedical cannabis use and cannabis use disorder: 2001–2002 and 2012–2013. Am J Psychiatry. 2020;177(7):611–8. 31964162 10.1176/appi.ajp.2019.19030284 PMC7332392

[38] Mannes ZL, Malte CA, Olfson M, Wall MM, Keyes KM, Martins SS, Increasing risk of cannabis use disorder among U.S. veterans with chronic pain: 2005–2019. Pain. 2023;164(9):2093–103. 37159542 10.1097/j.pain.0000000000002920 PMC10524371

[39] Bialas P, Böttge-Wolpers C, Fitzcharles MA, Gottschling S, Konietzke D, Juckenhöfel S, Cannabis use disorder in patients with chronic pain: Overestimation and underestimation in a cross-sectional observational study in 3 German pain management centres. Pain. 2023;164(6):1303–11. 36327134 10.1097/j.pain.0000000000002817

[40] Ditre JW, Brandon TH, Zale EL, Meagher MM. Pain, nicotine, and smoking: research findings and mechanistic considerations. Psychol Bull. 2011;137(6):1065–93. 21967450 10.1037/a0025544 PMC3202023

[41] Croghan IT, Hurt RT, Ganesh R, Bhagra O, Fischer KM, Vincent A, The association of current tobacco status with pain and symptom severity in fibromyalgia patients. Mayo Clin Proc Innov Qual Outcomes. 2021;5(3):614–24. 34195553 10.1016/j.mayocpiqo.2021.03.008 PMC8240153

[42] Larowe LR, Ditre JW. Pain, nicotine, and tobacco smoking: Current state of the science. Pain. 2020;161(8):1688–93. 32701828 10.1097/j.pain.0000000000001874 PMC8924914

[43] Zvolensky MJ, Mcmillan K, Gonzalez A, Asmundson GJG. Chronic pain and cigarette smoking and nicotine dependence among a representative sample of adults. Nicotine Tob Res. 2009;11(12):1407–14. 19828432 10.1093/ntr/ntp153 PMC2784489

[44] Sanderson E, Glymour MM, Holmes MV, Kang H, Morrison J, Munafò MR, Mendelian randomization. Nat Rev Methods Primers. 2022;2(1):1–21. 10.1038/s43586-021-00092-5 PMC7614635 37325194

[45] Koller D, Friligkou E, Stiltner B, Pathak GA, Løkhammer S, Levey DF, Pleiotropy and genetically inferred causality linking multisite chronic pain to substance use disorders. Mol Psychiatry. 2024;29(7):2021–30. 38355787 10.1038/s41380-024-02446-3 PMC11324857

[46] Deak JD, Zhou H, Galimberti M, Levey DF, Wendt FR, Sanchez-Roige S, Genome-wide association study in individuals of European and African ancestry and multi-trait analysis of opioid use disorder identifies 19 independent genome-wide significant risk loci. Mol Psychiatry. 2022;27(10):3970–9. 35879402 10.1038/s41380-022-01709-1 PMC9718667

[47] Toikumo S, Jennings MV, Pham BK, Lee H, Mallard TT, Bianchi SB, Multi-ancestry meta-analysis of tobacco use disorder identifies 461 potential risk genes and reveals associations with multiple health outcomes. Nat Hum Behav. 2024;8(6):1177–93. 38632388 10.1038/s41562-024-01851-6 PMC11199106

[48] Hatoum AS, Colbert SMC, Johnson EC, Huggett SB, Deak JD, Pathak GA, Multivariate genome-wide association meta-analysis of over 1 million subjects identifies loci underlying multiple substance use disorders. Nat Ment Health. 2023;1(3):210–23. 37250466 10.1038/s44220-023-00034-y PMC10217792

[49] Schaffer J, Fogelman N, Seo D, Sinha R. Chronic pain, chronic stress and substance use: Overlapping mechanisms and implications. Front Pain Res. 2023;4:1145934. 10.3389/fpain.2023.1145934 PMC10320206 37415830

[50] Johnson EC, Demontis D, Thorgeirsson TE, Walters RK, Polimanti R, Hatoum AS, A large-scale genome-wide association study meta-analysis of cannabis use disorder. Lancet Psychiatry. 2020;7(12):1032–45. 33096046 10.1016/S2215-0366(20)30339-4 PMC7674631

[51] Williams FMK, Elgaeva EE, Freidin MB, Zaytseva OO, Aulchenko YS, Tsepilov YA, Causal effects of psychosocial factors on chronic back pain: a bidirectional Mendelian randomisation study. Eur Spine J. 2022;31(7):1906–15. 35662366 10.1007/s00586-022-07263-2 PMC9273132

[52] All of Us Research Program Investigators; Denny JC, Rutter JL, Goldstein DB, Philippakis A, Smoller JW, The “All of Us” Research Program. N Engl J Med. 2019;381(7):668–76. 31412182 10.1056/NEJMsr1809937 PMC8291101

[53] Sudlow C, Gallacher J, Allen N, Beral V, Burton P, Danesh J, UK Biobank: An open access resource for identifying the causes of a wide range of complex diseases of middle and old age. PLoS Med. 2015;12(3):e1001779. 25826379 10.1371/journal.pmed.1001779 PMC4380465

[54] Loke MF, Wei H, Yeo J, Sng BL, Sia AT, Tan EC. Deep sequencing analysis to identify novel and rare variants in pain-related genes in patients with acute postoperative pain and high morphine use. J Pain Res. 2019;12:2755–70. 31571979 10.2147/JPR.S213869 PMC6756825

[55] O’Connor LJ, Schoech AP, Hormozdiari F, Gazal S, Patterson N, Price AL. Extreme polygenicity of complex traits is explained by negative selection. Am J Hum Genet. 2019;105(3):456–76. 31402091 10.1016/j.ajhg.2019.07.003 PMC6732528

[56] Smith AH, Jensen KP, Li J, Nunez Y, Farrer LA, Hakonarson H, Genome-wide association study of therapeutic opioid dosing identifies a novel locus upstream of OPRM1. Mol Psychiatry. 2017;22(3):346–52. 28115739 10.1038/mp.2016.257 PMC5407902

[57] Morris MC, Moradi H, Aslani M, Sun S, Karlson C, Bartley EJ, Haves and have-nots: Socioeconomic position improves accuracy of machine learning algorithms for predicting high-impact chronic pain. Pain. 2024. Available from: https://journals.lww.com/pain/fulltext/9900/haves_and_have_nots_socioeconomic_position.736.aspx. Accessed on 30 Mar 2025. 10.1097/j.pain.0000000000003451 PMC11985544 39451017

[58] Langer K, Wolf OT, Merz CJ, Jentsch VL. The effects of stress hormones on cognitive emotion regulation: A systematic review and integrative model. Neurosci Biobehav Rev. 2025;170:106040. 39909150 10.1016/j.neubiorev.2025.106040

[59] Sander D, Nummenmaa L. Reward and emotion: An affective neuroscience approach. Curr Opin Behav Sci. 2021;39:161–7.

[60] Hollon NG, Burgeno LM, Phillips PEM. Stress effects on the neural substrates of motivated behavior. Nat Neurosci. 2015;18(10):1405–12. 26404715 10.1038/nn.4114 PMC4721524

[61] Ashton H 5. Motivation. In: Brain function and psychotropic drugs. London (UK): Arnold; 2002. p. 83–104.

[62] Godoy LD, Rossignoli MT, Delfino-Pereira P, Garcia-Cairasco N, Umeoka EH de L. A comprehensive overview on stress neurobiology: Basic concepts and clinical implications. Front Behav Neurosci. 2018;12:127. 30034327 10.3389/fnbeh.2018.00127 PMC6043787

[63] Lewis RG, Florio E, Punzo D, Borrelli E. The Brain’s reward system in health and disease. Adv Exp Med Biol. 2021;1344:57–69. 34773226 10.1007/978-3-030-81147-1_4 PMC8992377

[64] Chrousos GP. Stress and disorders of the stress system. Nat Rev Endocrinol. 2009;5(7):374–81. 19488073 10.1038/nrendo.2009.106

[65] Vachon-Presseau E, Roy M, Martel MO, Caron E, Marin MF, Chen J, The stress model of chronic pain: evidence from basal cortisol and hippocampal structure and function in humans. Brain. 2013;136(3):815–27. 23436504 10.1093/brain/aws371

[66] Sinha R Chronic stress, drug use, and vulnerability to addiction. Ann N Y Acad Sci. 2008;1141(1):105–30. 18991954 10.1196/annals.1441.030 PMC2732004

[67] Méndez-Couz M, Manahan-Vaughan D, Silva AP, González-Pardo H, Arias JL, Conejo NM. Metaplastic contribution of neuropeptide Y receptors to spatial memory acquisition. Behav Brain Res. 2021;396:112887. 10.1016/j.bbr.2020.112864 32827566

[68] Kuhar MJ, Dall Vechia SE. CART peptides: Novel addiction- and feeding-related neuropeptides. Trends Neurosci. 1999;22(7):316–20. 10370256 10.1016/s0166-2236(98)01377-0

[69] Ezzatpanah S, Babapour V, Haghparast A. Differential contribution of orexin receptors within the ventral tegmental area to modulation of persistent inflammatory pain. Eur J Pain. 2016;20(7):1090–101. 26871274 10.1002/ejp.833

[70] Markovic T, Pedersen CE, Massaly N, Vachez YM, Ruyle B, Murphy CA, Pain induces adaptations in ventral tegmental area dopamine neurons to drive anhedonia-like behavior. Nat Neurosci. 2021;24(11):1601–13. 34663957 10.1038/s41593-021-00924-3 PMC8556343

[71] Gorwood P Neurobiological mechanisms of anhedonia. Dialogues Clin Neurosci. 2008;10(3):291–9. 18979942 10.31887/DCNS.2008.10.3/pgorwood PMC3181880

[72] Garfield JBB, Lubman DI, Yücel M. Anhedonia in substance use disorders: A systematic review of its nature, course and clinical correlates. Aust N Z J Psychiatry. 2014;48(1):36–51. 24270310 10.1177/0004867413508455

[73] Evrard HC, Craig AD. Insular cortex. In: Brain Mapping: An Encyclopedic Reference. Amsterdam (The Netherlands): Elsevier; 2015. p. 387–93.

[74] Pahng AR, Edwards S. The convergent neuroscience of affective pain and substance use disorder. Alcohol Res. 2021;41(1):14. 34976573 10.35946/arcr.v41.1.14 PMC8700315

[75] Campbell EJ, Lawrence AJ. It’s more than just interoception: The insular cortex involvement in alcohol use disorder. J Neurochem. 2021;157(5):1644–51. 33486788 10.1111/jnc.15310

[76] Jørgensen KN, Psychol C, Skjærvø I, Mørch-Johnsen L, Haukvik UK, Lange EH, Cigarette smoking is associated with thinner cingulate and insular cortices in patients with severe mental illness. J Psychiatry Neurosci. 2015;40(4):241–9. 25672482 10.1503/jpn.140163 PMC4478057

[77] Yeung EW, Craggs JG, Gizer IR. Comorbidity of alcohol use disorder and chronic pain: Genetic influences on brain reward and stress systems. Alcohol Clin Exp Res. 2017;41(11):1831–48. 29048744 10.1111/acer.13491 PMC5679730

[78] Bruijnzeel AW, Small E, Pasek TM, Yamada H. Corticotropin-releasing factor mediates the dysphoria-like state associated with alcohol withdrawal in rats. Behav Brain Res. 2010;210(2):288–91. 20193713 10.1016/j.bbr.2010.02.043 PMC3319063

[79] Méndez-Couz M, Manahan-Vaughan D, Silva AP, González-Pardo H, Arias JL, Conejo NM. Metaplastic contribution of neuropeptide Y receptors to spatial memory acquisition. Behav Brain Res. 2021;396:112887. 10.1016/j.bbr.2020.112864 32827566

[80] Calzà L Neuropeptides and receptors in Glia. In: Encyclopedia of Neuroscience. 2009:851–9.

[81] De Luca R, Nardone S, Grace KP, Venner A, Cristofolini M, Bandaru SS, Orexin neurons inhibit sleep to promote arousal. Nat Commun. 2022;13(1):1–15. 35851580 10.1038/s41467-022-31591-y PMC9293990

[82] Zhu Z, Chen G, He J, Xu Y. The protective effects of orexin B in neuropathic pain by suppressing inflammatory response. Neuropeptides. 2024;108:102458. 39255695 10.1016/j.npep.2024.102458

[83] Kajiyama S, Kawamoto M, Shiraishi S, Gaus S, Matsunaga A, Suyama H, Spinal orexin-1 receptors mediate anti-hyperalgesic effects of intrathecally-administered orexins in diabetic neuropathic pain model rats. Brain Res. 2005;1044(1):76–86. 15862792 10.1016/j.brainres.2005.03.007

[84] Cason AM, Smith RJ, Tahsili-Fahadan P, Moorman DE, Sartor GC, Aston-Jones G. Role of orexin/hypocretin in reward-seeking and addiction: Implications for obesity. Physiol Behav. 2010;100(5):419–28. 20338186 10.1016/j.physbeh.2010.03.009 PMC2886173

[85] Sharma R, Sharma A, Sahota P, Thakkar MM. Orexin gene expression is downregulated in alcohol dependent rats during acute alcohol withdrawal. Neurosci Lett. 2020;739:135416. 10.1016/j.neulet.2020.135347 33011195

[86] Matzeu A, Martin-Fardon R. Targeting the orexin system for prescription opioid use disorder: Orexin-1 receptor blockade prevents oxycodone taking and seeking in rats. Neuropharmacology. 2020;164:107906. 31841797 10.1016/j.neuropharm.2019.107906 PMC6954966

[87] Rasmussen K, White DA, Acri JB. NIDA’s medication development priorities in response to the Opioid Crisis: ten most wanted. Neuropsychopharmacology. 2019;44(4):657–9. 30538289 10.1038/s41386-018-0292-5 PMC6372702

[88] Sharp BM, Eisenstein TK, Madden JJ, Friedman H, editors. The Brain Immune Axis and Substance Abuse. New York (US): Plenum Press; 1995.

[89] Hassan Y, Esteves SC, Leyrer-Jackson JM, Thomas MP. The Neuroimmune System in Psychiatric Disorders. In: Neuroimmune Pharmacology and Therapeutics. Cham (Switzerland): Springer; 2024. p. 1025–60.

[90] Marchand F, Perretti M, McMahon SB. Role of the Immune system in chronic pain. Nat Rev Neurosci. 2005;6(7):521–32. 15995723 10.1038/nrn1700

[91] Raffaeli W, Malafoglia V, Bonci A, Tenti M, Ilari S, Gremigni P, Identification of MOR-positive B cell as possible innovative biomarker (Mu Lympho-Marker) for chronic pain diagnosis in patients with fibromyalgia and osteoarthritis diseases. Int J Mol Sci. 2020;21(4):1499. 32098316 10.3390/ijms21041499 PMC7073128

[92] Vasiliu O Current trends and perspectives in the immune therapy for substance use disorders. Front Psychiatry. 2022;13:882491. 35573367 10.3389/fpsyt.2022.882491 PMC9095939

[93] Prego-Domínguez J, Khazaeipour Z, Mallah N, Takkouche B. Socioeconomic status and occurrence of chronic pain: A meta-analysis. Rheumatology. 2021;60(3):1091–105. 33276382 10.1093/rheumatology/keaa758

[94] Mills SEE, Nicolson KP, Smith BH. Chronic pain: A review of its epidemiology and associated factors in population-based studies. Br J Anaesth. 2019;123(2):e273–e283. 31079836 10.1016/j.bja.2019.03.023 PMC6676152

[95] Lewis B, Hoffman L, Garcia CC, Nixon SJ. Race and socioeconomic status in substance use progression and treatment entry. J Ethn Subst Abuse. 2018;17(2):150–66. 28846065 10.1080/15332640.2017.1336959 PMC6125691

[96] Jordan KP, Thomas E, Peat G, Wilkie R, Croft P. Social risks for disabling pain in older people: A prospective study of individual and area characteristics. Pain. 2008;137(3):652–61. 18434022 10.1016/j.pain.2008.02.030

[97] Adams G, Salomons TV. Attending work with chronic pain is associated with higher levels of psychosocial stress. Can J Pain. 2021;5(1):107–16. 34189394 10.1080/24740527.2021.1889925 PMC8210861

[98] Elliott AM, Smith BH, Hannaford PC, Smith WC, Chambers WA. The course of chronic pain in the community: Results of a 4-year follow-up study. Pain. 2002;99(1–2):299–307. 12237208 10.1016/s0304-3959(02)00138-0

[99] Vogel M, Choi F, Westenberg JN, Cabanis M, Nikoo N, Nikoo M, Chronic pain among individuals experiencing homelessness and its interdependence with opioid and other substance use and mental illness. Int J Environ Res Public Health. 2022;19(1):5. 10.3390/ijerph19010005 PMC8751035 35010263

[100] Polcin DL. Co-occurring substance abuse and mental health problems among homeless persons: Suggestions for research and practice. J Soc Distress Homeless. 2015;25(1):1–10. 27092027 10.1179/1573658X15Y.0000000004 PMC4833089

[101] Lin C, Cousins SJ, Zhu Y, Clingan SE, Mooney LJ, Kan E, A scoping review of social determinants of health’s impact on substance use disorders over the life course. J Subst Use Addict Treat. 2024;166:209484. 39153733 10.1016/j.josat.2024.209484 PMC11418584

[102] Reboussin BA, Green KM, Milam AJ, Furr-Holden CDM, Ialongo NS. Neighborhood environment and urban African American marijuana use during high school. J Urban Health. 2014;91(6):1189–201. 25323775 10.1007/s11524-014-9909-0 PMC4242855

[103] Buu A, DiPiazza C, Wang J, Puttler LI, Fitzgerald HE, Zucker RA. Parent, family, and neighborhood effects on the development of child substance use and other psychopathology from preschool to the start of adulthood. J Stud Alcohol Drugs. 2009;70(4):489–98. 19515288 10.15288/jsad.2009.70.489 PMC2696289

[104] Maly A, Vallerand AH. Neighborhood, socioeconomic, and racial influence on chronic pain. Pain Manag Nurs. 2018;19(1):14–22. 29422123 10.1016/j.pmn.2017.11.004 PMC8895435

[105] Stanhope J, Breed MF, Weinstein P. Exposure to greenspaces could reduce the high global burden of pain. Environ Res. 2020;187:109641. 32447087 10.1016/j.envres.2020.109641 PMC7207132

[106] Jones CL, Haskin O, Younger JW. Association between chronic pain and fatigue severity with weather and air pollution among females with myalgic encephalomyelitis/chronic fatigue syndrome (ME/CFS). Int J Environ Res Public Health. 2024;21(12):1560. 39767402 10.3390/ijerph21121560 PMC11675267

[107] Szyszkowicz M Urban ambient air pollution and substance use disorder. Air Qual Atmos Health. 2022;15(6):1111–20.

[108] Berry MS, Rung JM, Crawford MC, Yurasek AM, Ferreiro AV, Almog S. Using greenspace and nature exposure as an adjunctive treatment for opioid and substance use disorders: Preliminary evidence and potential mechanisms. Behav Processes. 2021;186:104344. 33545317 10.1016/j.beproc.2021.104344 PMC9968503

[109] Díaz-Martínez F, Sánchez-Sauco MF, Cabrera-Rivera LT, Ortín-Fernández CA, Orenes-Piñero E, Ortega-García JA. Harnessing the healing power of nature: A review of natural interventions in substance abuse treatment and prevention. Environ Health Prev Med. 2024;29:64. 39537154 10.1265/ehpm.24-00145 PMC11570648

[110] Helgeson VS. Social support and quality of life. Qual Life Res. 2003;12(Suppl 1):25–31. 12803308 10.1023/a:1023509117524

[111] Che X, Cash R, Ng SK, Fitzgerald P, Fitzgibbon BM. A systematic review of the processes underlying the main and the buffering effect of social support on the experience of pain. Clin J Pain. 2018;34(11):1061–76. 29697476 10.1097/AJP.0000000000000624

[112] Cordero D The healing others: the essential role of social support on chronic pain management. Korean J Pain. 2024;37(3):280–9. 38946698 10.3344/kjp.24158 PMC11220379

[113] Yang C, Xia M, Zhou Y. How is perceived social support linked to life satisfaction for individuals with substance-use disorders? The mediating role of resilience and positive affect. Curr Psychol. 2020;41(5):2719–32.

[114] Pettersen H, Landheim A, Skeie I, Biong S, Brodahl M, Oute J, How social relationships influence substance use disorder recovery: A collaborative narrative study. Subst Abuse. 2019;13:1178221819833379. 30886519 10.1177/1178221819833379 PMC6410387

[115] Stevens E, Jason LA, Ram D, Light J. Investigating social support and network relationships in substance use disorder recovery. Subst Abus. 2015;36(4):396–9. 25259558 10.1080/08897077.2014.965870 PMC4375072

[116] Benville JR, Compton P, Giordano NA, Cheatle MD. Perceived social support in patients with chronic pain with and without opioid use disorder and role of medication for opioid use disorder. Drug Alcohol Depend. 2021;221:108619. 33667781 10.1016/j.drugalcdep.2021.108619 PMC8796693

[117] National Institute on Drug Abuse. Sex Differences in Substance Use Disorder Treatment. Available from: https://nida.nih.gov/publications/research-reports/substance-use-in-women/sex-differences-in-substance-use-disorder-treatment. Accessed on 25 Mar 2025.

[118] Williams EC, Fletcher OV, Frost MC, Harris AHS, Washington DL, Hoggatt KJ. Comparison of substance use disorder diagnosis rates from electronic health record data with substance use disorder prevalence rates reported in surveys across sociodemographic groups in the veterans health administration. JAMA Netw Open. 2022;5(6):e2219651. 35771574 10.1001/jamanetworkopen.2022.19651 PMC9247731

[119] Substance Abuse and Mental Health Services Administration. 2021 National Survey on Drug Use and Health (NSDUH) Releases. Available from: https://www.samhsa.gov/data/data-we-collect/nsduh-national-survey-drug-use-and-health/national-releases/2021#annual-national-report. Accessed on 25 Mar 2025.

[120] Kaiser Family Foundation. Substance Use Issues Are Worsening Alongside Access to Care. Available from: https://www.kff.org/policy-watch/substance-use-issues-are-worsening-alongside-access-to-care/. Accessed on 25 Mar 2025.

[121] Mereish EH. Substance use and misuse among sexual and gender minority youth. Curr Opin Psychol. 2019;30:123–7. 31202102 10.1016/j.copsyc.2019.05.002 PMC6859198

[122] Rustøen T, Wahl AK, Hanestad BR, Lerdal A, Paul S, Miaskowski C. Gender differences in chronic pain—findings from a population-based study of Norwegian adults. Pain Manag Nurs. 2004;5(3):105–17. 15359222 10.1016/j.pmn.2004.01.004

[123] Munce SEP, Stewart DE. Gender differences in depression and chronic pain conditions in a national epidemiologic survey. Psychosomatics. 2007;48(5):394–9. 17878497 10.1176/appi.psy.48.5.394

[124] Zelaya CE, Dahlhamer JM, Lucas JW, Connor EM. Chronic pain and high-impact chronic pain among U.S. adults, 2019. 2020. Available from: https://stacks.cdc.gov/view/cdc/97308. Accessed on 30 Mar 2025. 33151145

[125] Grossman ER, Benjamin-Neelon SE, Sonnenschein S. Alcohol consumption during the COVID-19 pandemic: A cross-sectional survey of US adults. Int J Environ Res Public Health. 2020;17(24):9189. 33316978 10.3390/ijerph17249189 PMC7763183

[126] Koopmann A, Georgiadou E, Kiefer F, Hillemacher T. Did the general population in germany drink more alcohol during the COVID-19 pandemic lockdown? Alcohol Alcohol. 2020;55(6):698–9. 32556079 10.1093/alcalc/agaa058 PMC7337704

[127] Koller D, Pathak GA, Wendt FR, Tylee DS, Levey DF, Overstreet C, Epidemiologic and genetic associations of endometriosis with depression, anxiety, and eating disorders. JAMA Netw Open. 2023;6(1):e2251214. 36652249 10.1001/jamanetworkopen.2022.51214 PMC9856929

[128] Grotzinger AD, Rhemtulla M, de Vlaming R, Ritchie SJ, Mallard TT, Hill WD, Genomic structural equation modelling provides insights into the multivariate genetic architecture of complex traits. Nat Hum Behav. 2019;3(5):513–25. 30962613 10.1038/s41562-019-0566-x PMC6520146

[129] All of Us Research Program Investigators; Denny JC, Rutter JL, Goldstein DB, Philippakis A, Smoller JW, The “All of Us” Research Program. N Engl J Med. 2019;381(7):668–76. 31412182 10.1056/NEJMsr1809937 PMC8291101

[130] Taylor AMW, Becker S, Schweinhardt P, Cahill C. Mesolimbic dopamine signaling in acute and chronic pain: Implications for motivation, analgesia, and addiction. Pain. 2016;157(6):1194–8. 26797678 10.1097/j.pain.0000000000000494 PMC4866581

[131] Zorrilla EP, Logrip ML, Koob GF. Corticotropin releasing factor: A key role in the neurobiology of addiction. Front Neuroendocrinol. 2014;35(2):234–44. 24456850 10.1016/j.yfrne.2014.01.001 PMC4213066

[132] Wise RA, Robble MA. Dopamine and addiction. Annu Rev Psychol. 2020;71:79–106. 31905114 10.1146/annurev-psych-010418-103337

[133] Kang X, Tang H, Liu Y, Yuan Y, Wang M. Research progress on the mechanism of orexin in pain regulation in different brain regions. Open Life Sci. 2021;16(1):46–54. 33817297 10.1515/biol-2021-0001 PMC7874592

[134] Aliev F, De Sa Nogueira D, Aston-Jones G, Dick DM. Genetic associations between orexin genes and phenotypes related to behavioral regulation in humans, including substance use. Mol Psychiatry. 2025;1–9. 39880903 10.1038/s41380-025-02895-4 PMC12185332

[135] Huhn AS, Dunn KE. The orexin neurotransmitter system as a target for medication development for opioid use disorder. Neuropsychopharmacology. 2024;49(1):329–30. 37491670 10.1038/s41386-023-01679-0 PMC10700298

[136] John WS, Wu LT. Chronic non-cancer pain among adults with substance use disorders: Prevalence, characteristics, and association with opioid overdose and healthcare utilization. Drug Alcohol Depend. 2020;209:108466. 10.1016/j.drugalcdep.2020.107902 PMC7127943 32088587

[137] Zale EL, LaRowe LR, Boissoneault J, Maisto SA, Ditre JW. Gender differences in associations between pain-related anxiety and alcohol use among adults with chronic pain. Am J Drug Alcohol Abuse. 2019;45(5):479–87. 30864852 10.1080/00952990.2019.1578968

[138] Stennett B, Anderson MB, Vitus D, Ferguson E, Dallery J, Alappattu M, Sex moderates the effects of experimentally induced musculoskeletal pain on alcohol demand in healthy drinkers. Drug Alcohol Depend. 2021;219:108423. 10.1016/j.drugalcdep.2020.108475 PMC8911397 33385694

